# Minimizing complications following transinguinal preperitoneal modified Kugel mesh herniorrhaphy: a double blind prospective randomized clinical trial

**DOI:** 10.1038/s41598-022-20803-6

**Published:** 2022-09-30

**Authors:** Heng-Chieh Chiang, Jesun Lin, Jian-Ting Chen, Yu-Chi Hsu, Pao-Hwa Chen

**Affiliations:** 1 Department of Surgery, Division of Urology, Changhua Christian Hospital, 135, Nanxiao St., Changhua City, Changhua County 500 Taiwan; 2 Department of Chemical Engineering, Chung Yuan Christian University, Taoyuan, Taiwan

**Keywords:** Anatomy, Gastroenterology, Medical research, Urology

## Abstract

Transinguinal preperitoneal (TIPP) single-layer mesh herniorrhaphy has been proven effective. Mesh manufacturers make either a single-unit, two-layer mesh design or a separate optional onlay with the pre-peritoneal mesh. For peace of mind, most surgeons still incorporate the optional onlay. This study evaluated any counterproductive effects of adding the onlay to single-layer TIPP mesh herniorrhaphy and compared the long-term efficacy. This prospective, single-surgeon, single-center, randomized trial compared two groups of 50 consecutive patients at a 1 to 1 ratio. The control group received a single-layer modified Kugel mesh in the preperitoneal space, while the study group received the optional onlay mesh in the inguinal canal with preperitoneal mesh placement. A single surgeon performed the same operation to place the preperitoneal mesh in both groups, the only difference being the placement of the optional onlay mesh in the study group. A blinded researcher performed post-operative interviews using a series of questions at 1, 3, 6, and 12 months after surgery, and another unblinded researcher organized and performed statistical analysis of the peri-operative and post-operative data. The primary endpoints included foreign body sensation, pain, and any other discomfort in the inguinal region following surgery; and the secondary endpoints included recurrence and any complications related to surgery. The patient demographics were similar between the two groups. The average follow-up period was 29 months. Two patients in the 1-layer group and one patient in the 2-layer group were lost to follow-up. Postoperative pain, numbness and soreness were similar between groups. No patients experienced a foreign body sensation after 3 months in the 1-layer group, while five patients still had a foreign body sensation at 12 months in the 2-layer group. No recurrence was noted in either group during the follow-up period. Adequate dissection of the preperitoneal space is the key to a successful single-layer TIPP herniorrhaphy. With decreased materials in the inguinal canal, single-layer TIPP has a lower rate of long-term postoperative discomfort without increasing the risk of future recurrence.

Trial registration: ISRCTN 47111213

## Introduction

Before the introduction of polypropylene mesh, tissue repair was the main form of herniorrhaphy, but a poor long-term efficacy (> 10% recurrence in 10 years) and immediate postoperative pain were the main drawbacks^1,2^. The invention of polypropylene mesh improved the recurrence rate but increased post-herniorrhaphy chronic pain (> 3 months). Due to simplicity, Lichtenstein mesh repair is easy to learn, but post-herniorrhaphy chronic pain is a major drawback, with some studies reporting an incidence of up to 40%^3,4^. Explanations for the high rate of chronic herniorrhaphy pain include periosteum damage during mesh positioning, nerve damage (due to stretching, division, compression, electrocautery injury) or inflammation from surrounding mesh materials^3–6^. Careful nerve dissection and manipulation during inguinal canal preparation before mesh placement can help to reduce the chance of chronic pain, but mesh placed in the vicinity of inguinal canal nerves can cause inflammation, fibrosis and granuloma formation^7–9^. Mesh manufacturers either make a single-unit, two-layer design (i.e., UHS and PHS mesh by Ethicon US, LLC) or a preperitoneal mesh with a separate optional onlay mesh to strengthen the posterior wall (i.e., PerFix Plug by Davol Inc. or ULTRAPRO Plug by Ethicon US, LLC). Some surgeons performing transinguinal preperitoneal (TIPP) mesh repair routinely place the optional onlay in the inguinal canal for fear of future recurrence. In our retrospective study, we noted that 1-layer preperitoneal mesh placement did not put patients at risk of future recurrence (< 1%). The key to TIPP mesh placement is adequate dissection of the preperitoneal space, which results in flat mesh placement covering the hernia defects. Most recent studies have compared different types of mesh and different mesh placements. In this prospective study, we investigated whether the use of the optional onlay mesh included in the modified Kugel mesh is associated with long-term complications and examined the efficacy of single-layer TIPP herniorrhaphy in terms of preventing recurrence.

## Methods/design

### Ethics and informed consent

The institutional review board at Changhua Christian Hospital approved the trial protocol (IRB number 140312) of this study. The trial was registered online with ISRCTN (online registry number: 47111213 assigned on: 2017/03/21). In the preoperative outpatient clinic, all participants were informed in detail about the method of randomization and surgical method before informed consent was obtained according to the IRB protocol.

### Study population

The study population included patients undergoing elective inguinal hernia surgery using MK mesh at Changhua Christian Hospital. The exclusion criteria included: (1) recurrent hernia, (2) femoral hernia, (3) hernia defects larger than the Posiflex® memory ring diameter, and (4) patients refusing to participate in the randomization protocol. Of the 110 patients eligible during the study period, seven patients did not meet the inclusion criteria and three patients refused to participate in the study. One-hundred patients were allocated into two groups, with 50 patients in each group. During the follow-up period, two patients in the 1-layer group and two in the 2-layer group were lost to follow-up due to changes in telephone number.

### Study endpoints

This randomized, prospective clinical trial aimed to investigate the reduction in post-operative complications associated with mesh herniorrhaphy and determine the long-term effectiveness of single-layer preperitoneal mesh placement as compared with two-layer. The primary endpoint was to evaluate patients’ chronic postoperative complications (chronic pain, neuropathy, foreign body sensation). The secondary objective was to determine the overall success of the hernia operation (recurrence, perioperative and early postoperative complications).

### Study design, patient randomization and blinding

**Figure 1:**
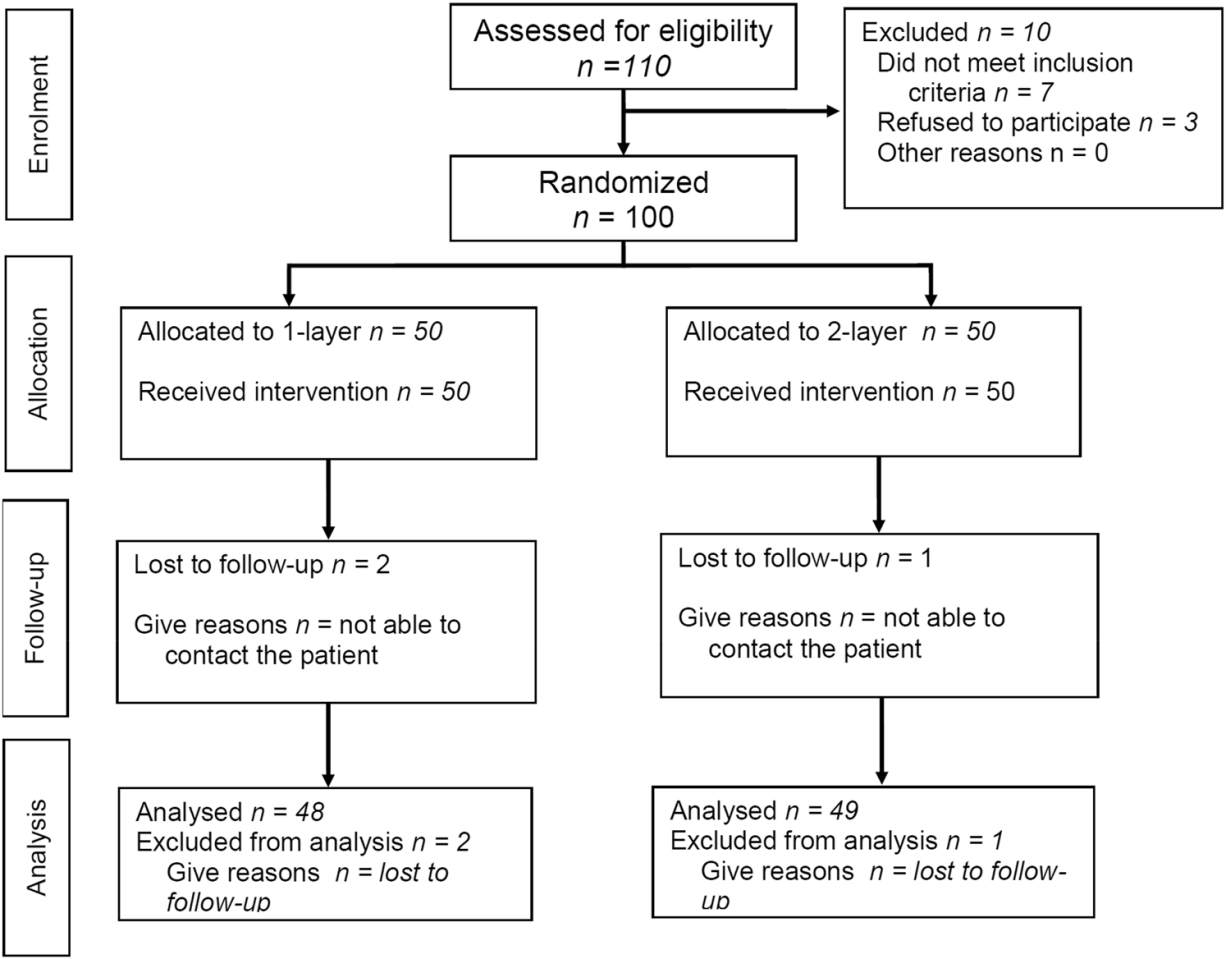
CONSORT flow diagram.

**Figure 2:**
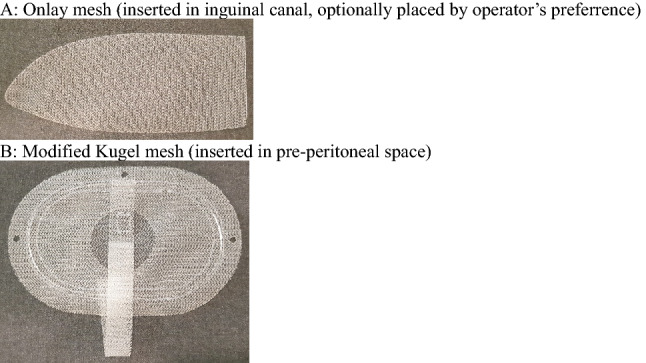
Modified Kugel mesh packaging and content.

This was a prospective, double-blind (patients and observer blinded), single-center, randomized study with a two-arm parallel-group design. A series of 100 consecutive patients undergoing elective inguinal hernia surgery were randomized by computer-generated allotment into two groups at a 1:1 ratio (Fig. 1). A research fellow (P.H.C.) handled data collection and concealment of randomization. Once a patient was diagnosed with inguinal hernia at the outpatient clinic, they were assigned a patient number (1–100), which was used in the computer-generated allotment. The patients were blinded as to whether they would receive 1-layer or 2-layer herniorrhaphy. To eliminate bias in surgical techniques, a single surgeon (H.C.C.) performed all the hernia operations during this trial. Before the start of the surgery, the research fellow (P.H.C.) informed the surgeon (H.C.C.) as to whether the patient was assigned to the 1- or 2-layer group. A modified Kugel (MK) 8 × 12 cm mesh (with Posiflex Memory Technology, BARD-Davol Inc., Cranston, RI, USA) was used in this study. The standard packaging comes with an MK mesh and an optional onlay mesh (Fig. 2). The manufacturer recommends using the optional onlay mesh if the hernia defect extends beyond the Posiflex® ring. The control group (1-layer) received a single-layer MK mesh in the preperitoneal space. The study group (2-layer) received the optional onlay patch in the inguinal canal in addition to the preperitoneal placement of the MK mesh. All patients attended a routine outpatient clinic follow-up on postoperative day 7 (POD-7) to assess any early post-op complications. As the surgeon (H.C.C.) did not participate in the follow-up period, a blinded research assistant (Y.C.H.) performed telephone interviews on all patients using a set of questions at regular intervals (at the 1st, 3rd, 6th and 12th months, and every 6 months subsequently post-op). The patient recruitment period ran from 2014/05/22 to 2015/06/30. All patients were followed-up for a minimum duration of 1 year after their operation. The follow-up period ranged from 2014/05/22 to 2017/06/30.

### Surgical method

**Figure 3:**
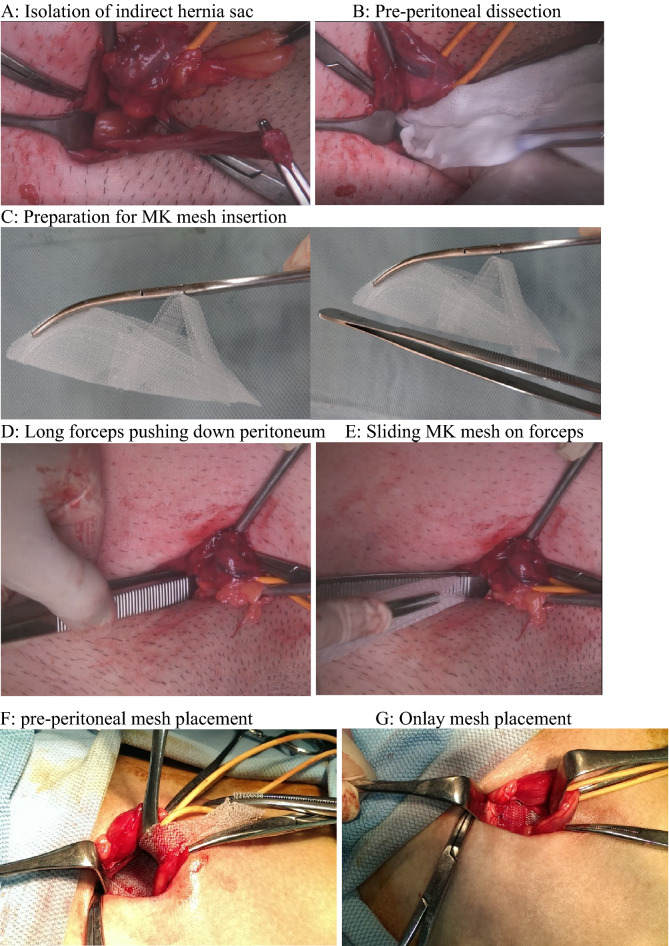
Surgical technique and intra-operative view. Operation on right side indirect hernia (EHS: P-L-1).

Depending on patient preference and existing contraindications, all patients received either spinal or general anesthesia. After completion of the anesthesia, prophylactic antibiotics (cefazolin 1000 mg) were given at least 30 min prior to incision. The operative field was shaved with electric clippers and disinfected with 2% chlorhexidine + 75% ethanol (Easy Antiseptic liquid 2%, Panion & BF Biotech Inc, Taipei, Taiwan). Operative time was calculated using the digital operating room timer and was recorded from the initial skin incision until the last suture securing the mesh. The surgical method, preperitoneal dissection and mesh placement were as described in our previous retrospective study^10^. Intraoperative findings, such as depth of subcutaneous fat (distance from the skin to the fascia of the external oblique muscle), perispermatic cord lipomas, nerves identified in the inguinal canal, and type of hernia defect (Nyhus classification) were recorded. After the flat placement of the MK mesh and securing of the positioning strap, the digital timer was stopped, and the time was recorded (Fig. 3). After the placement of the 1st layer, the surgeon was informed if the patient set to receive 1 layer or 2 layers. For the 1-layer group, the fascia and wound were closed with 2–0 and 4–0 braided absorbable sutures (Polysorb, Covidien, USA). For patients randomized into the 2-layer group, a second timer was started to record the time needed for placement of the onlay mesh, and then the wound was closed using the same method. Unless the patient had renal function impairment, routine oral 25 mg diclofenac potassium (Cataflam, Novartis Pharmaceuticals) every 8 h and intravenous nalbuphine hydrochloride 10 mg (Bain, Genovate Biotechnology Co.) every 6 h when necessary were used for postoperative pain management. For patients with renal function impairment, oral 500 mg acetaminophen tablets every 6 h were used instead of diclofenac potassium.

### Data collection (long-term patient follow-up and evaluation)

After the initial post-operative clinical examination, a blinded research assistant (Y.C.H.) followed-up with the patients via a series of telephone interviews. A set of questions (Table 1) was designed to serve as a history-taking lesson. If any patients exhibited signs of possible recurrence, they were asked to return to the clinic for a physical examination and imaging (ultrasound and CT scan) to confirm the diagnosis of recurrence. Post-operative complications such as recurrence, pain, foreign body sensation, and infections were recorded and analyzed in the two groups. Our classification of foreign body sensation was any discomfort not described as pain, soreness, or numbness, or any discomfort that deviated from the norm that affected daily activities. The Clavien-Dindo classification for post-operative complications was used to evaluate the severity of the complications. Unless the patient was lost during follow-up, all patients were followed-up for a minimum of 12 months. All gathered data were then passed to the research fellow (P.H.C.) for statistical analysis.

**Table 1:** Questionnaires used during telephone interview.

Question 1	Since the last visit/interview, have you experienced a bulging appearance in the groin region during your daily activities? (If no, skip to question 4.)
Question 2	Does your job require heavy lifting? What other physical activity do you participate in on a daily basis?
Question 3	Do you notice a bulging mass or groin pain when you stand up, lift heavy objects, strain, or cough?
Question 4	Have you sought another doctor’s advice or received surgical treatment for the bulging mass or groin pain?
Question 5	Have you experienced any pain in the groin, scrotum, or abdomen area? (Assess pain with VAS score)
Question 6	Is there anything that will make the pain worse or better?
Question 7	If the pain is persistent, have you needed to seek medical advice for the pain? Was pain medication prescribed?

### Sample size and statistical analysis

All relevant results in the two groups were analyzed using 1-way ANOVA, the chi-square test, linear regression and the Kruskal–Wallis test with IBM SPSS statistics version 22 software, with a *p*-value < 0.05 considered statistically significant. Post-operative discomfort and pain after 3 months was reported in 40% of patients^11^ who received mesh herniorraphy with onlay mesh. We estimated a postoperative rate of less than 10% for single-layer TIPP mesh herniorrhaphy from reported studies and our retrospective study^10,12–16^. With an α of 0.05 and a power of 0.8, the sample size was estimated to be 47 in each group (total 94 patients). With an expected dropout rate of 10 to 15%, the study was initially estimated to require 110 patients randomized into two groups (Fig. 1).

## Results

**Figure 4:**
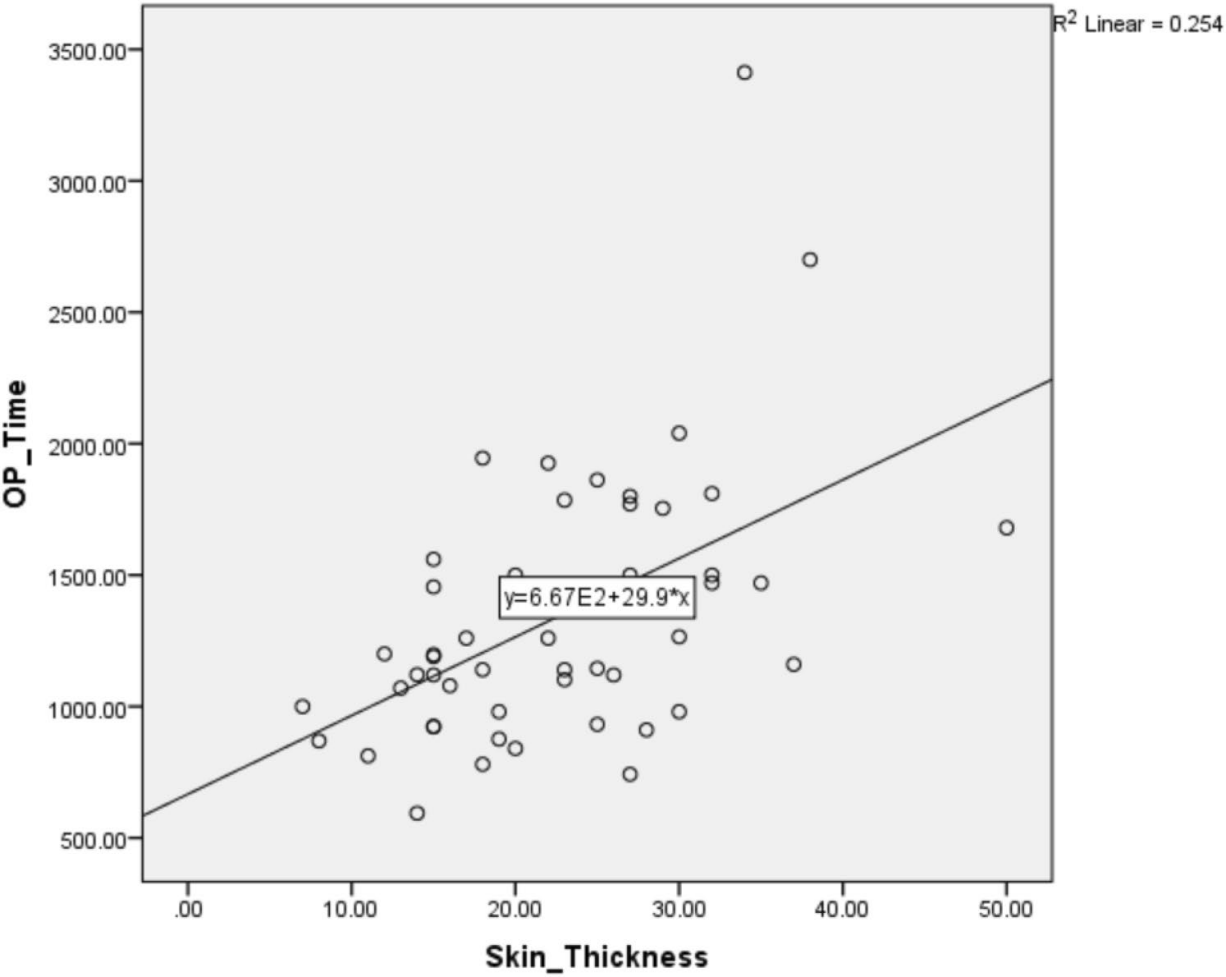
Correlation between Skin thickness and OP time (Skin_thickness: mm, OP_Time: seconds).

The patients’ general information, peri-operative and in-hospital data are presented in Tables 2 and 3. There were no discrepancies in terms of age, sex, form of anesthesia, body mass index (BMI), EHS classification, time needed to place mesh in the preperitoneal space, length of stay, or visual analog scale (VAS) score on discharge between the two groups. Without requiring time to place the extra onlay, the single-layer group had a shorter total operative time (22.4 vs. 29.5 min, *p* < 0.001). During long-term follow-up, two patients in the 1-layer and one patient in the 2-layer group were lost. The average onlay placement time was 451 s (7 min 30 s). Upon reviewing the recorded data, we noted that the average duration for onlay placement in the initial 10 cases was10.9 min, with a range of 10–15 min. After the initial 10 cases, the average onlay placement duration decreased to 6.7 min, with a range of 3–10 min. Increased repetition most likely contributed to the decrease in the onlay placement duration. Pain, soreness and numbness complications were similar between the two groups (Table 4). At the initial post-op day 7 and 1-month follow-up, both groups had very similar numbers and rates of pain, soreness and numbness complications. At the 3-, 6- and 12-month follow-ups, the numbers and percentages of pain, soreness and numbness in the 2-layer group were higher, but these results were not statistically significant (*p* > 0.5). The number of reported foreign body sensation occurrences was slightly lower in the 1-layer group from POD-7 to the 3-month follow-up, but this was not statistically significant (Table 5, P > 0.3). The 1-layer group had a noticeable decrease in the rate of reported foreign body sensation at the 6- and 12-month follow-ups (*n* = 0, *p* = 0.043, *p* = 0.023, respectively). Although both groups had similar rates of spinal anesthesia and patients with benign prostatic hyperplasia requiring medication, the 2-layer group had a higher rate of post-operative urine retention needing catheterization (N = 2 vs. 8, P = 0.045). When analyzing different variables and the relation to operative time, the experience of the assistant (different years of resident training or surgical technician) and EHS classification were not related to an increased operative time. There was a linear correlation between operative time and skin thickness (Fig. 4, *p* < 0.05). There was no recurrence in either group.

**Table 2:** Patients basic information.

	1 layer	2 layers	P-value
	n = 50	n = 50	
Age (years)	61	63	0.374
Male/Female	48/2	49/1	0.557
Spinal anesthesia	21 (42%)	22 (44%)	0.840
BMI	23.97	24.41	0.483
Length of stay (days)	2	2	0.405
VAS score ≥ 3 on discharge	8	5	0.372
Post-OP urine retention	2 (4%)	8 (16%)	**0.045**
Lost follow-up Mean follow-up months (range)	2 (4%) 31.92 (24–39)	1 (2%) 32.32 (24–37)	0.558 0.640

Significant values are in bold.

**Table 3:** Intra-operative finding.

	1 layer	2 layers	P-value
	n = 50	n = 50	
**Laterality**			
Right	28 (56)	18 (36)	0.045
Left	15 (30%)	31 (62%)	0.001
Bilateral	7 (14%)	1 (2%)	0.027
**EHS classification**			
L-1	18 (36%)	19 (38%)	0.836
L-2	11 (22%)	9 (18%)	0.617
M-X	21 (42%)	22 (44%)	0.840
Subcutaneous thickness (skin to ext aponeurosis, mm)	23	25	0.084
**Assistant experience**			
Resident	17 (34%)	14 (28%)	0.437
Fellow	8 (16%)	6 (12%)	0.564
Surgical Tech	25 (50%)	30 (60%)	0.315
**Mean OP time (min)**			
TIPP mesh	22.4	22.0	0.787
On-lay mesh	0	7.5	**0.001**
Total OP time	22.4	29.5	**0.001**

Significant values are in bold.

**Table 4:** Post-operative complains (pain, soreness, numbness).

	1 layer	2 layer	P
	n = 50	n = 50	value
**POD-7**			0.511
None	47 (94%)	46 (92%)	
Pain	2 (4%)	1 (2%)	
Numbness	1 (2%)	3 (6%)	
Soreness	0 (0%)	0 (0%)	
**1st month F/U**			0.318
None	36 (75%)	34 (7%)	
Pain	4 (8%)	4 (8%)	
Numbness	6 (13%)	11 (22%)	
Soreness	2 (4%)	0 (0%)	
**3rd month F/U**			0.622
None	34 (71%)	36 (73%)	
Pain	5 (10%)	3 (6%)	
Numbness	8 (17%)	10 (20%)	
Soreness	1 (2%)	0 (0%)	
**6th month F/U**			0.647
None	43 (90%)	43 (88%)	
Pain	2 (4%)	2 (4%)	
Numbness	2 (4%)	4 (8%)	
Soreness	1 (2%)	0 (0%)	
** > 12th month F/U**			0.508
None	46 (96%)	46 (94%)	
Pain	0 (0%)	1 (2%)	
Numbness	1 (2%)	2 (4%)	
Soreness	1 (2%)	0 (0%)

**Table 5:** Post-operative foreign body sensation.

	1 layer	2 layers	P
	n = 50	n = 50	value
POD-7	4 (8%)	7 (14%)	0.337
1st month	6 (13%)	8 (16%)	0.592
3rd month	4 (8%)	4 (8%)	0.976
6th month	0 (0%)	4 (8%)	**0.043**
> 12th month	0 (0%)	5 (10%)	**0.023**

Significant values are in bold.

## Discussion

The emphasis of earlier hernia repair studies has been on lowering the operative time, future recurrence rate, wound infection rate, and rates of other morbidities (i.e., incarceration, testicular problems)^2,15^. Tissue repair lacks long-term efficacy, with a reported recurrence rate of more than 10% at the 10-year follow-up point^2^, while mesh repair has a recurrence of less than 5%^1,11^. During our follow-up period (range, 24–39 months), we did not treat any patients for recurrence, and saw no signs of suspected recurrence that needed additional clinical hours for physical examination to confirm the diagnosis. In our previous retrospective experience and clinical experience, one recurrence was noted, and TEP was used to repair the hernia protrusion^10,17^. In that recurrence case, inadequate preperitoneal dissection resulted in suboptimal mesh placement^17–19^. We have since standardized our dissection with 7 wet surgical gauzes, and no recurrence was noted in the time (2012–2017) spanning from the previous study to this study^10^. Surgical plane disruption in anterior TIPP mesh herniorrhaphy is a concern regarding future recurrence. Prevention of recurrence should be the priority, with adequate preperitoneal dissection, identification of lipoma and occult peritoneal reflection being important aspects in prevention^10,17–19^. Pre-operative imaging can help identify recurrence and also identify other anatomical anomalies (i.e., lipoma)^20,21^. In our experience, with recurrence in anterior preperitoneal mesh placements (< 1% of cases), either the anterior or laparoscopic approach are feasible depending on the surgeon’s preference and experience.

With a longer follow-up period, the focus of recent studies has shifted towards evaluating post-operative quality of life (QoL) and minimizing long-term post-operative pain/discomfort^1,9,11,22–31^. Surrogate end points such as post-operative discomfort (i.e., pain, foreign body sensation, etc.) have been used to quantify post-operative QoL. Even though the short form-36 (SF-36) questionnaire has been used to evaluate QoL in different diseases and treatments, few hernia studies have incorporated SF-36 to quantify post-operative QoL. Iftikhar et al. used SF-36 to show that post-operative physical and emotional function improved after hernia surgery^31^. Currently lacking an inguinal hernia-specific QoL questionnaire, Huang et al. proposed the HERQL questionnaire, which aimed to assess post-herniorrhaphy QoL; this needs to be validated in future studies^32^.

With all the advantages of the Lichtenstein method (i.e., short operative time, low recurrence rate), the debilitating effect of post-herniorrhaphy chronic pain is a major drawback^1,11,25–28^. Many factors can influence post-operative pain, such pre-operative pain, intra-operative nerve manipulation/damage, and mesh-related inflammatory processes^6,23,29,30,33–35^. Pre-operative pain and discomfort are good indicators of post-operative discomfort^34,35^. Mier et al. used the SF-12 questionnaire and pre-operative visual analog SPS score to determine post-operative QoL^35^. In that study, patients with SPS ≤ 12 had an improved long-term QoL. Manangi et al. showed that 27% of patients without pre-operative pain developed chronic pain, while 77% with pre-operative pain developed chronic pain^34^. Wright et al. studied the reasons for pain in inguinal hernia patients at the histological level, and found that compression neuropathy of the inguinal nerve is a culprit for pre-operative pain. On a microscopic level, increased pain is correlated with an increased nerve diameter, fascicle count, and more myxoid material within the perineurium and endoneurium^36^. These factors are all innate to patients and can only be identified during surgery. Some intraoperative factors that surgeons can be wary of include nerve identification/manipulation and minimizing foreign materials around the nerves. Some studies have suggested that placement of the mesh in the pre-peritoneal space while minimizing nerve involvement (i.e., nerve manipulation and damage) are related to a decrease in the rate of chronic herniorrhaphy pain^6,13,14,27,30,37–39^. After implanting prosthetic material, the body will start to undergo acute inflammation and subsequent chronic fibroblastic change, which lead to scarring and fibrosis. In some cases, the inflammatory process can also cause damage to the surrounding tissues^7,8^. Nienhuijs et al. compared the Prolene Hernia System (PHS), mesh plug, and Lichtenstein methods for open hernia repair, and found that there was no difference in postoperative pain (39.7% at 3 months and 43.3% long-term) between the three groups^11^. One possible culprit for the high rate of post-operative pain is the presence of foreign materials in the inguinal canal near the inguinal nerves. In a study by Nienhuijs et al., the rate of chronic pain increased from 39 to 43%, which might suggest the presence of “late-onset chronic pain”. Chronic pain is usually defined as post-operative pain for longer than 6 months, while late-onset chronic pain is usually initially asymptomatic but increasingly more symptomatic after 6 months. Reinpold et al. reported the nerve management of all different open herniorrhaphy procedures with a follow-up duration of at least 5 years^6^. The study concluded that neurolysis, nerve contact with mesh and the Lichtenstein method are related to the development of chronic herniorrhaphy pain^6^. The study also stated the need to differentiate between short-term and long-term chronic pain in future studies. The hypothesis of the cause of late persistent chronic pain is the presence of foreign materials in the inguinal canal, causing progressive chronic inflammatory irritation and fibrotic changes leading to nerve traction. In our study, the rate of neuropathy (pain, soreness, numbness) at 3-months post-op was similar in both groups, but the complaint of a foreign body sensation was statistically significantly different (no patients in the single-layer group complained of this sensation at the 6-month follow-up point). An additional patient complained of a foreign body sensation at 12 months in the 2-layer group. We hypothesized that chronic inflammation might be the reason for the increase in the number of patients experiencing a foreign body sensation in the 2-layer group.

The first limitation in our study is the initial sample size. The reported postoperative pain/discomfort for MK mesh with onlay is not available, therefore we used the 40% reported rate in Lichtenstein studies. In assuming the rate of 40%, the sample size would be underpowered. Second limitation would be the telephone interview as form of follow-up. In our current clinical practice and healthcare environment, telephone interviews are the most effective way of following these patients for periods of more than 3 months. The follow-up period is also a limitation to determine any late recurrence patients. Single surgeon design of this study is a limitation but also maintain the same quality of surgeon throughout the study period. The single surgeon design limits the generalizability of the study to surgeons with different experience and training.

## Conclusions

In this randomized, prospective study, we demonstrated that 1-layer preperitoneal mesh placement is equally effective as 2-layer placement, with a similar recurrence rate. In eliminating the use of onlay mesh, we decreased the manipulation and implantation of foreign material in the inguinal canal. The decreased foreign material also led to a decrease in foreign body sensation (0% after 6 months) and an improved post-operative QoL. Therefore, we strongly believe that properly-placed, single-layer TIPP MK mesh provides the advantage of open mesh repair without the drawback of chronic herniorrhaphy pain associated with Lichtenstein repair.

## Abbreviations

MK: Modified Kugel
POD: Postoperative day
TIPP: Transinguinal preperitoneal
